# Supplementary material for: Wnt/β-Catenin Inhibition Disrupts Carboplatin Resistance in Isogenic Models of Triple-Negative Breast Cancer
Source: Front Oncol. 2021 Jul 22;11:705384. doi: 10.3389/fonc.2021.705384 (PMC8340846; doi:10.3389/fonc.2021.705384)
Supplement: Supplementary Table 3 — Primer List. [file Table_3.docx]

| **Target** | **For/Rev** | **Sequence** |
| --- | --- | --- |
| AXIN 2 | FOR | AGTGCAAACTTTCGCCAACC |
|  | REV | TGAAGGACCTGTATCCACTGTC |
| cMYC | FOR | TGAGGAGACACCGCCCAC |
|  | REV | CAACATCGATTTCTTCCTCATCTTC |
| OCT4 | FOR | GTGGAGGAAGCTGACAACAA |
|  | REV | ATTCTCCAGGTTGCCTCTCA |
| NANOG | FOR | ACAACTGGCCGAAGAATAGCA |
|  | REV | GGTTCCCAGTCGGGTTCAC |
| SOX2 | FOR | TTGCTGCCTCTTTAAGACTAGGA |
|  | REV | CTGGGGCTCAAACTTCTCTC |
| RPL19 | FOR | AGTATGCTCAGGCTTCAGAAGA |
|  | REV | ATTGGTCTCATTGGGGTCTAAC |
| GAPDH | FOR | TCAAGAAGGTGGTGAAGCAGG |
|  | REV | ACCAGGAAATGAGCTTGACAAA |
| LGR5 | FOR | CTTACGTCACTGATGGTGCTTGC |
|  | REV | CTTGGAGAAAGAGATTTAGCCAGG |
| CTNNB1 | FOR | CCCACTAATGTCCAGCGTTT |
|  | REV | AACGCATGATAGCGTGTCTG​ |
