# Supplementary figures and images for: Wnt/β-Catenin Inhibition Disrupts Carboplatin Resistance in Isogenic Models of Triple-Negative Breast Cancer
Source: Front Oncol. 2021 Jul 22;11:705384. doi: 10.3389/fonc.2021.705384 (PMC8340846; doi:10.3389/fonc.2021.705384)

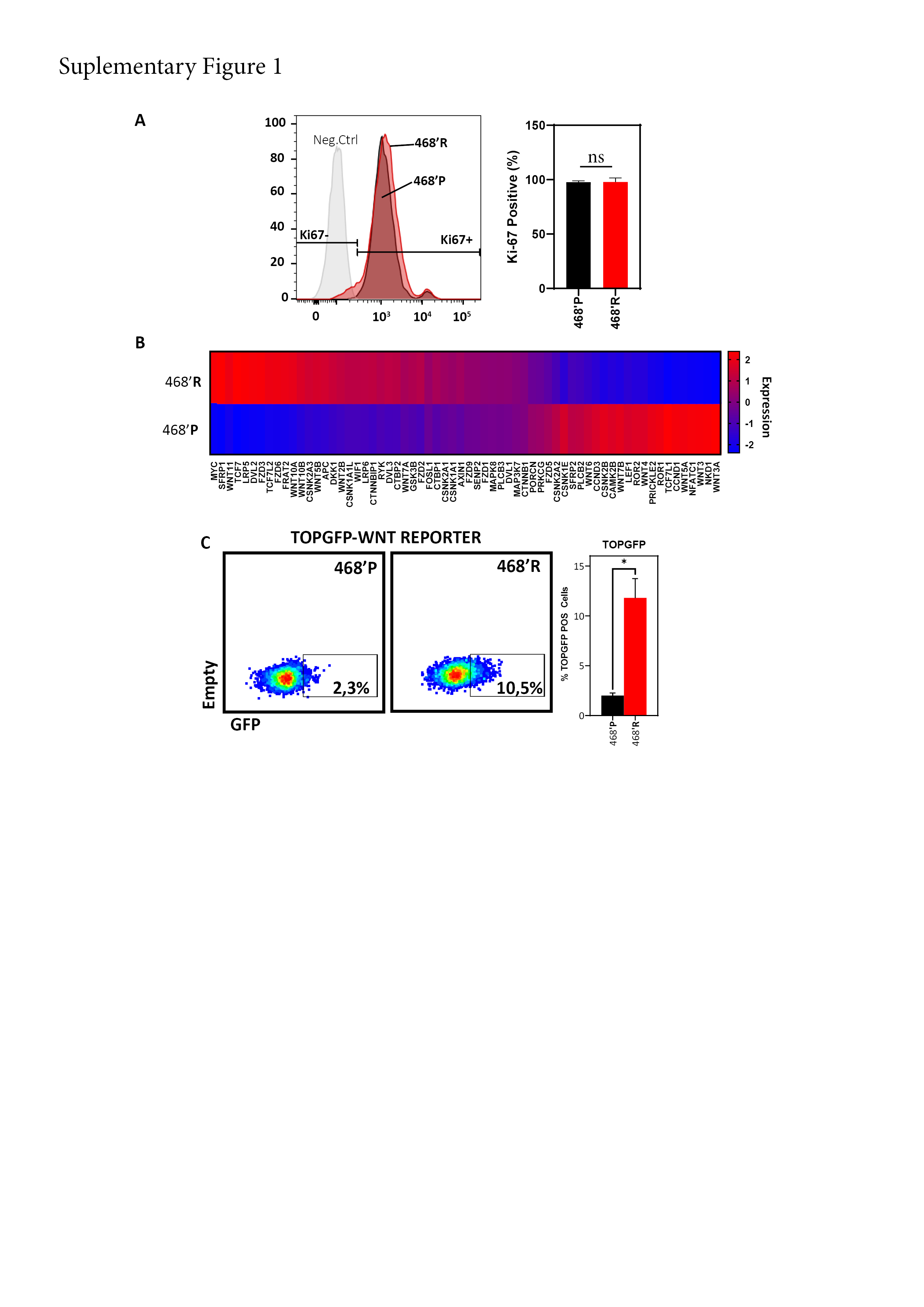

Supplement: Supplementary Figure 1 — (A) Representative histogram of flow cytometry Ki67 frequency in 468’P and 468’R cells (left) and statistical analysis (n=3). (B) Expression heatmap of Wnt-related genes from the WNT Pathway Pluripotency cluster from Figure 1F . (C) Flow cytometry analysis of TOPGFP-Wnt reporter activity in 468’P and 468’R cells (n=3). (Barplots represent mean + SEM. *p <0.05, **p<0.01, ***p<0.001, ****p<0.0001, ns= non significant). [file Image_1.jpeg]

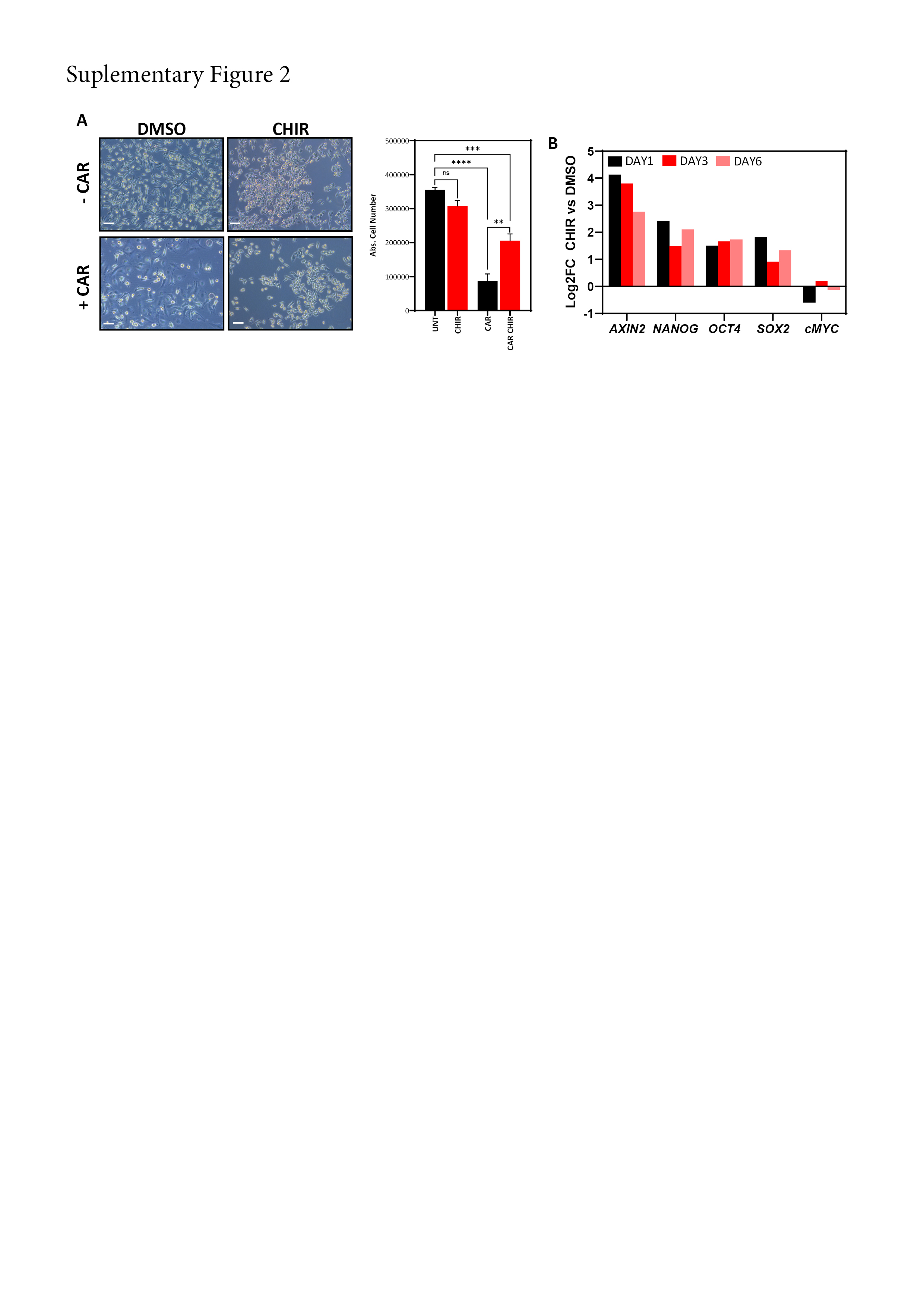

Supplement: Supplementary Figure 2 — (A) Phase-contrast microscopy (left, scale bar: 100 μm) of MDA-MB-231 cells treated for 72 hours with 35 μM Carboplatin in the presence or absence of 4 μM CHIR and statistical analysis of mean absolute cell numbers (n=4). One-way ANOVA with correction for multiple comparisons using the Holm-Sidak method. (B) mRNA level fold change (Log2) Wnt target AXIN2, and stem cell markers in MDA-MB-231 cells treated with 4 μM CHIR vs. DMSO (n=1, average of technical replicates). (Barplots represent mean + SEM. *p <0.05, **p<0.01, ***p<0.001, ****p<0.0001, ns= non significant). [file Image_2.jpeg]

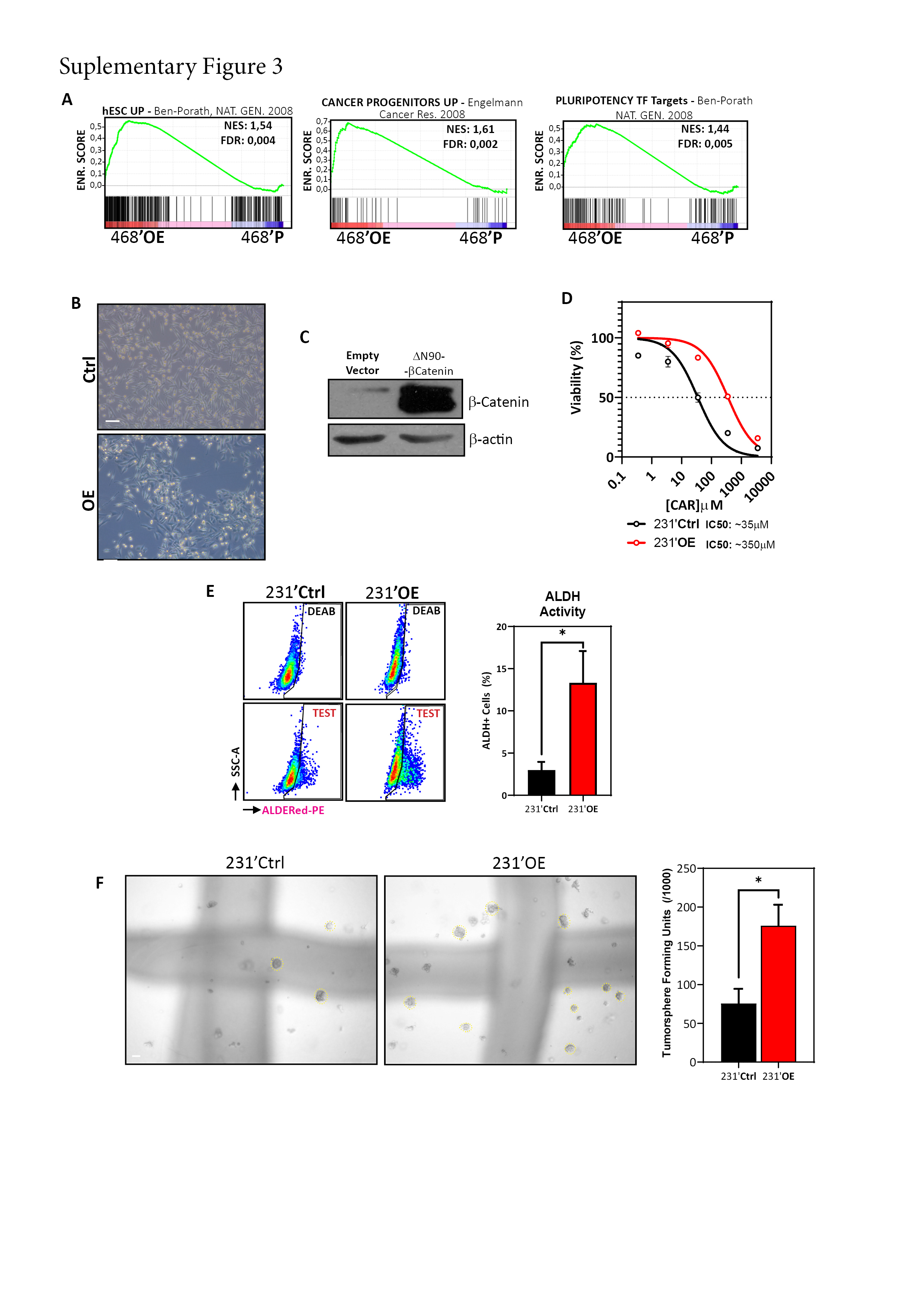

Supplement: Supplementary Figure 3 — (A) GSEA of hESCs (26) (left), Cancer Progenitor (46) (center), and pluripotency transcription target gene sets (26) (right) in 468’OE vs 468’P cells. (B) Phase-contrast microscopy (scale bar: 100 μm) of control MDA-MB-23 and 231’OE (Δn90 β-catenin overexpression). (C) Western blot of total β-catenin in MDA-MB-231 cells transduced with an empty vector or truncated, constitutively active β-catenin isoform ΔN90. β-actin was used as a loading control. (D) Non-linear fit model of [CAR] vs. normalized response for IC50 determination (right). (n=2) (E) Representative scatterplots of flow cytometric analysis of aldehyde dehydrogenase activity (left) and statistical analysis of the mean percentage of ALDH+ cells in 231’OE and 231’Ctrl cells using Welch’s t-test (n=5) (right). (F) Representative brightfield images of tumorspheres generated from 231’Ctrl and 231’OE cells (left, scale bar: 100 μm) and statistical analysis of mean tumorsphere forming units (number of spheres/number of seeded single cells) (right; n=3). Welch’s t-test. (Barplots represent mean + SEM. *p <0.05, **p<0.01, ***p<0.001, ****p<0.0001, ns = non significant). [file Image_3.jpeg]

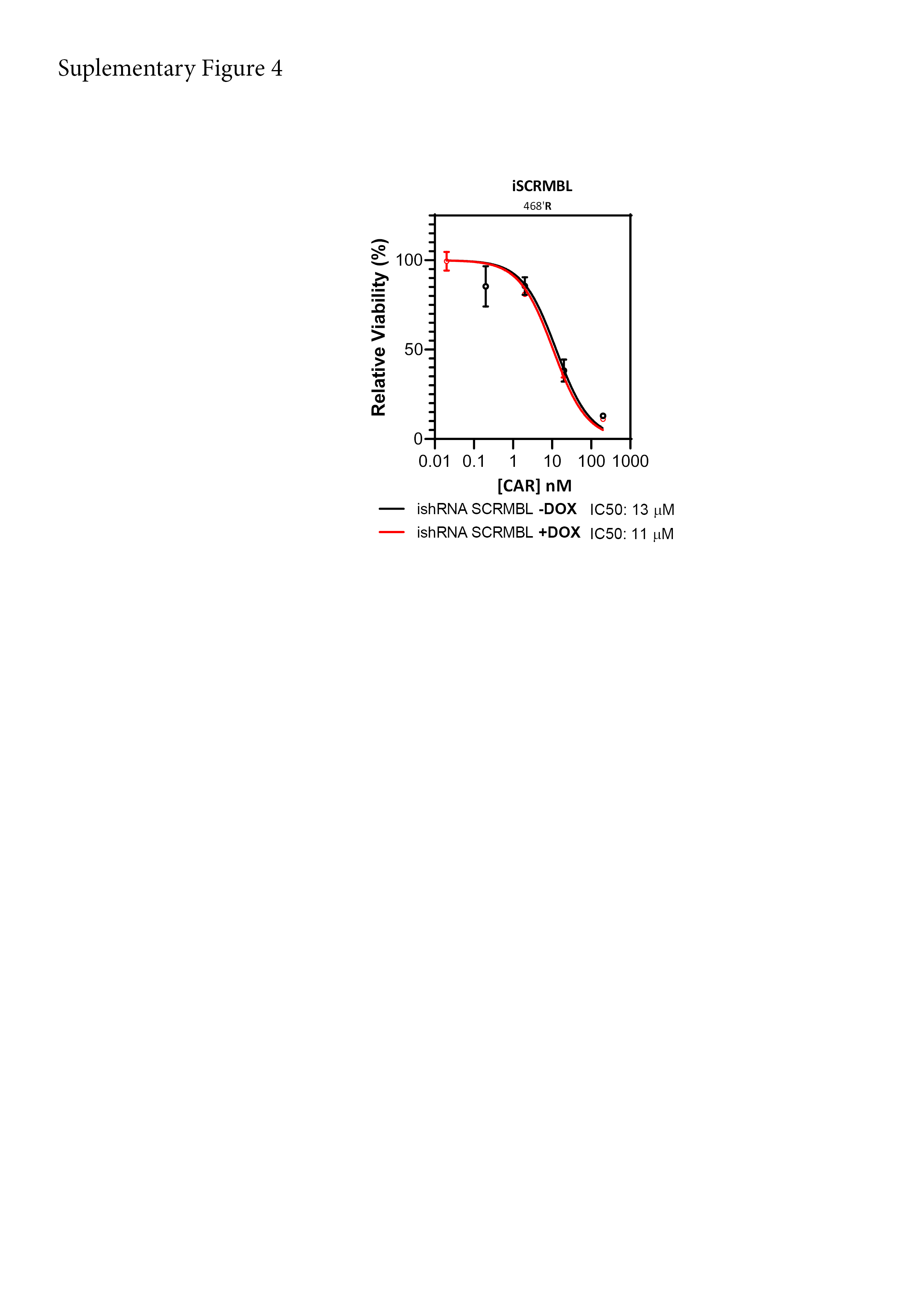

Supplement: Supplementary Figure 4 — Non-linear fit model of [CAR] vs. normalized response for IC50 determination (right) in 468’R cells transduced with inducible scrambled non-targeting shRNAs in the presence or absence of DOX. (n=2). [file Image_4.jpeg]

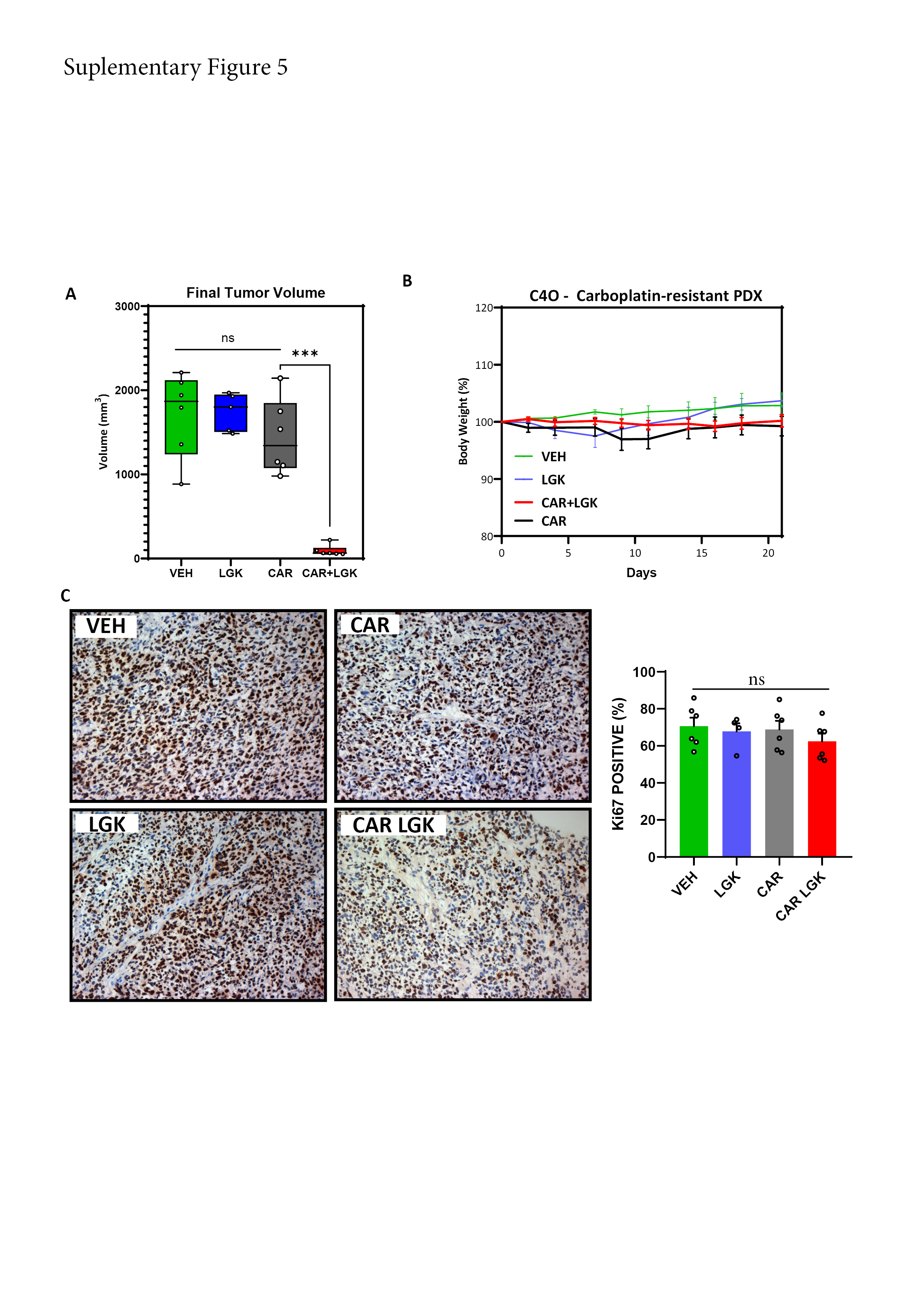

Supplement: Supplementary Figure 5 — (A) Final tumor volumes of VEH (n=6), LGK (n=5), CAR (n=6) and CAR+LGK (n=6) treated mice. One way-ANOVA with Holm-Sidak correction for multiple comparisons. (B) Bodyweight change in percentage of initial treatment bodyweight for VEH (n=6), LGK (n=5), CAR (n=6) and CAR+LGK (n=6) treated mice. No statistically significant changes detected by two way ANOVA. (C) Representative brightfield microscopy (20x magnification) of tumor sections labeled with anti-human Ki67 (left) and corresponding statistical analysis of the mean frequency of Ki67 positive cells. (VEH, CAR, CAR+LGK n=6 and LGK n=4). One-way ANOVA with Holm-Sidak correction for multiple comparisons. (Barplots represent mean + SEM. *p <0.05, **p<0.01, ***p<0.001, ****p<0.0001, ns = non significant). [file Image_5.jpeg]
